# Expression of Concern: Necroptosis is a key pathogenic event in human and experimental murine models of non-alcoholic steatohepatitis

**DOI:** 10.1042/CS20140732_EOC

**Published:** 2026-10-14

**Authors:** Marta B. Afonso, Pedro M. Rodrigues, Tânia Carvalho, Marta Caridade, Paula Borralho, Helena Cortez-Pinto, Rui E. Castro, Cecília M.P. Rodrigues

**Affiliations:** 1 Research Institute for Medicines (iMed.ULisboa), Faculty of Pharmacy, Universidade de Lisboa, Av. Prof. Gama Pinto, 1649-003 Lisbon, Portugal; 2 Histology and Comparative Pathology Laboratory, Instituto de Medicina Molecular, Av. Prof. Egas Moniz, Edificio Egas Moniz, 1649-028 Lisbon, Portugal; 3 ESTEsL, Av. D. João II, 1990-096 Lisbon, Portugal; 4 Instituto de Anatomia Patológica, Universidade de Lisboa, Av. Prof. Egas Moniz, 1649-035 Lisbon, Portugal; 5 Hospital Cuf Descobertas, R. Mário Botas, Parque das Nações, 1998-018 Lisbon, Portugal; 6 Department of Gastroenterology, Hospital Santa Maria, Faculty of Medicine, Universidade de Lisboa, Av. Prof. Egas Moniz, 1649-035 Lisbon, Portugal

**Keywords:** chronic liver diseases, mixed lineage kinase domain-like (MLKL), non-alcoholic steatohepatitis (NASH), necroptosis, oxidative stress, receptor-interacting protein 3 (RIP3)

The Editorial Office has been made aware of potential issues surrounding the scientific validity of this paper is hence issuing an expression of concern to notify readers.

Some of the data presented in the manuscript has subsequently been reused in several other publications, with the original data not being appropriately cited. Specifically, this includes the following:

- Figure 1A Control H&E panel has been reused in the Figure 1A Control H&E panel in Afonso et al. 2016 (DOI: 10.1038/cddis.2016.280)
- Figure 1B Control RIP3 panel has been reused in the Figure 1A Control RIP3 panel in Afonso et al. 2016 (DOI: 10.1038/cddis.2016.280)
- Figure 3A HFCD 6wk H&E panel has been reused in the Figure 1 HFCD 6 Weeks panel in Cortez-Pinto et al. 2016 (DOI: 10.1016/j.jpge.2016.01.004)
- Figure 6B WT MCD 2 wk H&E panel has been reused in the Figure 1b WT MCD 2 wks H&E panel in Rodrigues et al. 2017 (DOI: 10.1038/cddis.2017.172)

The authors have been contacted for comment and have confirmed that the data published in this manuscript was generated to support multiple complementary studies and was therefore reused across the subsequent publications highlighted without appropriate cross-referencing. They have indicated that these issues are limited to the selection of representative histological and staining images and, as such, do not compromise the scientific validity, robustness, or interpretation of the underlying experiments.

The other relevant journals were contacted but did not respond. As such, the data remains reused and not appropriately cited. In order to notify readers, this Expression of Concern will therefore remain published permanently or until appropriate citation is added to the other articles in question.

